# A Medicaid Statewide Hypertension Quality Improvement Project: Initial Results

**DOI:** 10.7759/cureus.36132

**Published:** 2023-03-14

**Authors:** Shari D Bolen, Siran Koroukian, Jackson T Wright, Harry Persaud, Douglas Einstadter, Jordan Fiegl, Adam T Perzynski, Douglas Gunzler, Catherine Sullivan, Jonathan Lever, Michael Konstan, Dushka Crane, Allison Lorenz, Michelle Menegay, Doug Spence, Arun RajanBabu, Wendy Groznik, Tonni Oberly, Xiaokun Qian, Christopher R Jordan, Phyllis Virgil, Sinead Yarberry, Emily Saunders, Alice M Teall, Joyce Zurmehly, Melissa Nance, Stephen Albanese, Donald Wharton, Mary S Applegate

**Affiliations:** 1 Center for Health Care Research and Policy, The MetroHealth Medical Center, Cleveland, USA; 2 Medicine, Case Western Reserve University School of Medicine, Cleveland, USA; 3 Population and Quantitative Health Sciences, Case Western Reserve University School of Medicine, Cleveland, USA; 4 Population Health and Care Management, Better Health Partnership, Cleveland, USA; 5 Pediatrics, Case Western Reserve University School of Medicine, Cleveland, USA; 6 Public Health, The Ohio Colleges of Medicine Government Resource Center, Columbus, USA; 7 Quality Improvement, Chris R. Jordan LLC, Cincinnati, USA; 8 Quality Improvement, Phyllis Virgil LLC, Washington DC, USA; 9 Nursing, Ohio State University College of Nursing, Columbus, USA; 10 Quality Improvement, Ohio Department of Medicaid, Columbus, USA; 11 Family Medicine, Ohio Department of Medicaid, Columbus, USA; 12 Internal Medicine-Pediatrics, Ohio Department of Medicaid, Columbus, USA

**Keywords:** primary healthcare services, racial equity, health services research, quality improvement projects, systemic hypertension

## Abstract

Background

Hypertension control is critical to reducing cardiovascular disease, challenging to achieve, and exacerbated by socioeconomic inequities. Few states have established statewide quality improvement (QI) infrastructures to improve blood pressure (BP) control across economically disadvantaged populations. In this study, we aimed to improve BP control by 15% for all Medicaid recipients and by 20% for non-Hispanic Black participants.

Methodology

This QI study used repeated cross-sections of electronic health record data and, for Medicaid enrollees, linked Medicaid claims data for 17,672 adults with hypertension seen at one of eight high-volume Medicaid primary care practices in Ohio from 2017 to 2019. Evidence-based strategies included (1) accurate BP measurement; (2) timely follow-up; (3) outreach; (4) a standardized treatment algorithm; and (5) effective communication. Payers focused on a 90-day supply (vs. 30-day) of BP medications, home BP monitor access, and outreach. Implementation efforts included an in-person kick-off followed by monthly QI coaching and monthly webinars. Weighted generalized estimating equations were used to estimate the baseline, one-year, and two-year implementation change in the proportion of visits with BP control (<140/90 mm Hg) stratified by race/ethnicity.

Results

For all practices, the percentage of participants with controlled BP increased from 52% in 2017 to 60% in 2019. Among non-Hispanic Whites, the odds of achieving BP control in year one and year two were 1.24 times (95% confidence interval: 1.14, 1.34) and 1.50 times (1.38, 1.63) higher relative to baseline, respectively. Among non-Hispanic Blacks, the odds for years one and two were 1.18 times (1.10, 1.27) and 1.34 times (1.24, 1.45) higher relative to baseline, respectively.

Conclusions

A hypertension QI project as part of establishing a statewide QI infrastructure improved BP control in practices with a high volume of disadvantaged patients. Future efforts should investigate ways to reduce inequities in BP control and further explore factors associated with greater BP improvements and sustainability.

## Introduction

Hypertension control is critical to reducing cardiovascular morbidity and mortality [1-4], yet achieving blood pressure (BP) control is challenging, especially in economically disadvantaged populations [5-7]. One successful model for accelerating evidence-based strategies from randomized trials into practice has been through quality improvement (QI) efforts within health systems or regional collaboratives, as demonstrated by the Veterans Administration and Kaiser Permanente [8-13]. In a recent effort with a regional health improvement collaborative from Northeast Ohio, a positive deviance approach was effective in accelerating the translation of hypertension best practices into care for the region leading to improved BP control and reduced racial and income inequities in BP control [14].

Beyond health systems and regional efforts, less work has been done to achieve statewide improvements in BP control. Building on regional efforts [14], we partnered with payers, medical schools, and primary care clinics to develop a statewide model for QI, with an initial focus on hypertension. Although regional and statewide efforts for hypertension improvement may have similar tasks around engagement, collaboration, data sharing, audit and feedback, and QI coaching, the practical partners and activities needed to establish a statewide model (versus a regional model) will differ given the large geographic area. One initial statewide and then multistate initiative by Egan et al. [15] assisted primary care champions in receiving a hypertension certificate, provided BP data feedback to practices, and promoted evidence-based strategies for hypertension control. While successful, this model did not initially engage with payers and is only one potential model. Given the diversity across states, providing additional models for collaborative statewide improvement efforts for hypertension control that engage payers and other stakeholders could result in a greater ability to impact hypertension control across the United States.

Between 2012 and 2015, the rate of hypertension control (BP ≤140/90 mmHg) for Medicaid enrollees in the state of Ohio was less than 50%. To address the low rate of hypertension control, the Ohio Department of Medicaid (ODM) established two separately funded but aligned initiatives: (1) a unique statewide cardiovascular health collaborative (Ohio Cardiovascular Health Collaborative; Cardi-OH) [16] primarily focused on the dissemination of cardiovascular evidence-based best practices (described further elsewhere) [17]; and (2) a statewide QI infrastructure with a pilot quality improvement project (QIP) in high-volume Medicaid practices for paired implementation, aimed at improving hypertension control and addressing inequities in BP control. In this paper, we describe the establishment of the statewide QI infrastructure and present the initial results of the statewide hypertension QIP from eight high-volume Medicaid primary care practices.

## Materials and methods

Study design and population

This study evaluates the impact of a QIP on BP control using linked Medicaid claims and repeated cross-sections of electronic health record (EHR) data over time. This study was approved by the Institutional Review Board at Case Western Reserve University (CWRU) (protocol number: 2018-2248). The study population consisted of individuals 18 years of age or older diagnosed with hypertension and seeking care at one of eight high-volume Medicaid primary care practices where the QIP was implemented (n = 17,672).

Statewide QI infrastructure and QIP formation

The Statewide QI Infrastructure and Hypertension QIP was a collaboration among ODM, the Medicaid Managed Care Plans (MCPs), the Ohio Colleges of Medicine Government Resource Center (GRC), QI consultants using the Institute for Healthcare Improvement’s (IHI) model for improvement, academic team members from the Ohio schools of medicine with Case Western Reserve University serving as the lead, and eight high-volume Medicaid primary care practices recruited as a convenience sample from diverse geographic areas across the state. The determination of roles within the organizations took time to develop, especially because some organizations had overlapping areas of expertise leading to competition around roles, such as EHR data extraction and visualization and QI coaching. As the funder, ODM made the final determinations related to organizations and roles (Table 1). One adaptation occurred in January 2019 related to QI coaching. GRC initially provided two QI coaches for the practices; however, this was transitioned to the Ohio State University (OSU) College of Nursing graduate program to support the development of advanced practice nurses (APNs) and was overseen by GRC, the QI consultant, and two OSU APN program directors.

**Table 1:** Organization roles and activities for the statewide QI infrastructure. IHI = Institute for Healthcare Improvement; MCP = Managed Care Plans; QI = quality improvement; OSU = Ohio State University; EHR = electronic health record; QIP = quality improvement project

Organization	Role	Description of activities
Ohio Department of Medicaid	Funder the Steer project in conjunction with partner organizations. Manage MCP engagement	Led monthly meetings with MCPs to align QI activities with primary care practice QI activities. Steering Committee meetings weekly
Ohio State Government Resource Center	Project management. QI coaching to practices through 2018 EHR data extraction and visualization. Practice recruitment	Obtained legal agreements for data sharing from practices. Developed aggregated and clinic-level control charts from data. Assist with practice recruitment QI coaching
QI consultants (IHI trained)	QI consulting for overall project	Guidance to QI coaches. Led QI didactics within monthly webinars for practices and MCPs
Academic Medical Centers	Academic expertise in hypertension and social drivers of health EHR data extraction expertise. Primary care practice engagement and recruitment. QI coaching transitioned to Ohio State University College of Nursing in January 2019	Development of change package with evidence-based best practices for hypertension control. Practice recruitment. Development of data elements and queries for QIP. QI coaching
Primary care practices	Conduct QI around hypertension care by testing elements of the change package. Partner with payers	Test the statewide QI infrastructure by piloting the hypertension QIP
Medicaid MCPs	Collaborative QI activities with practices. Reduce payer-level barriers to hypertension care	Test the statewide QI infrastructure by piloting the hypertension QIP

Medicaid MCPs were engaged as active participants in the QI efforts for their enrollees. The project aligned with the Centers for Medicare and Medicaid Services (CMS) requirements for Medicaid MCPs to participate in performance improvement projects over two-year cycles. While MCPs are mandated to conduct QI projects in two-year cycles by CMS, they also receive financial incentives for achieving quality metric goals, including BP. Primary care practices also received financial incentives through the various value-based payment models where BP was a quality metric. Patients were not directly involved in the formal QIP infrastructure; however, both MCPs and primary care clinics engaged with patients to better understand barriers to hypertension care as part of the QI activities (e.g., calling patients as they created Pareto charts around barriers to follow-up). Several clinics also had patients as part of their QI teams or boards although this was not a requirement to participate.

**Figure 1:**
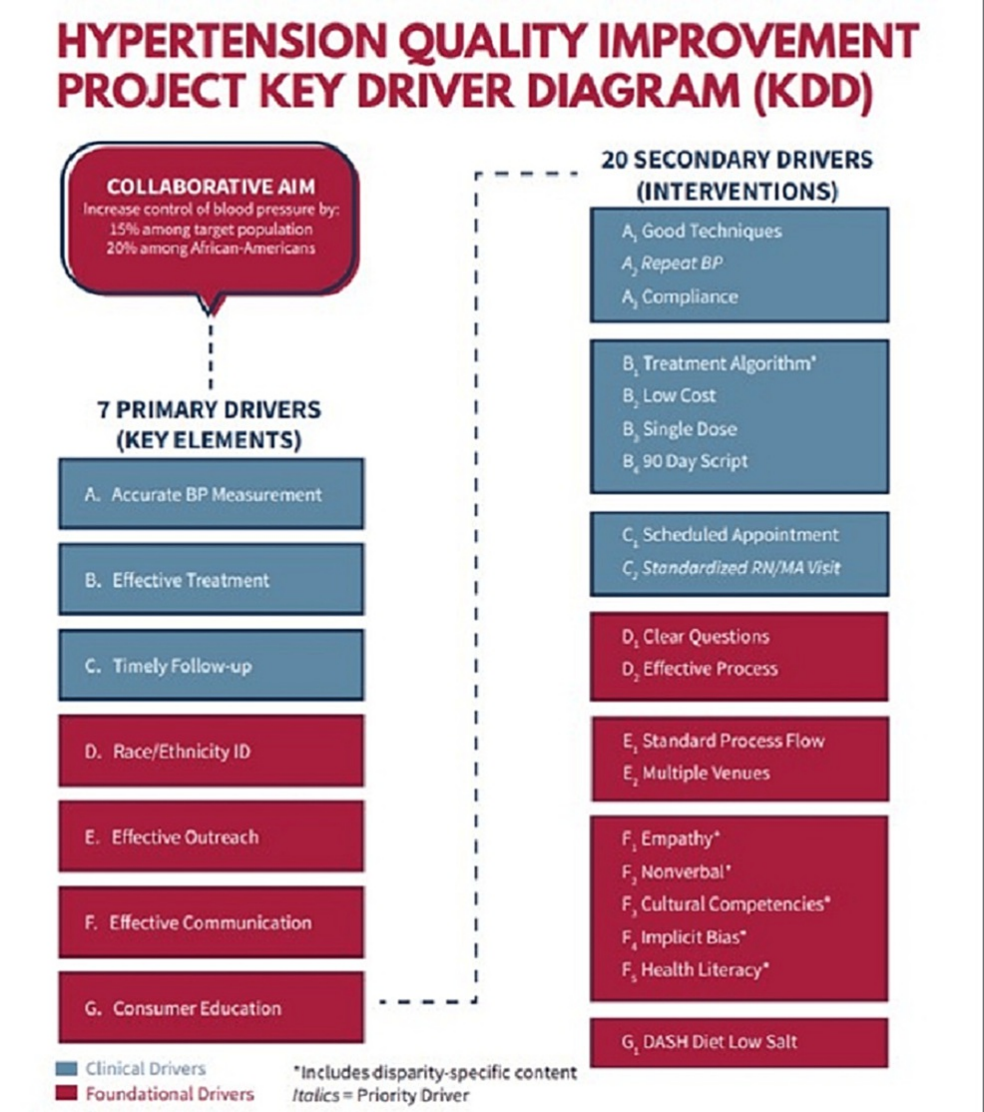
Hypertension quality improvement project key driver diagram. ID = identification; BP = blood pressure; DASH = dietary approaches to stop hypertension; KDD = key driver diagram

The planning period for the project lasted six months and included (1) the determination of roles and trust building among the organizations; (2) the development of the change package, including a key driver diagram (Figure 1) and specific, measurable, achievable, realistic, and timely (SMART) aims; (3) consensus on data elements to be used for process and outcome measures as well as testing of the EHR data query; (4) development of the implementation strategy described below; (5) practice recruitment; and (6) developing and executing legal agreements for data sharing. We aimed to recruit at least two practice champions per practice (ideally a prescribing provider and staff member) although we encouraged all practice team members to attend the QIP kickoff (a one-day meeting to start the project), monthly QI coaching meetings, and monthly webinars called Action Period calls. We collected baseline data from January 1, 2017, to October 31, 2017. The QIP implementation started on November 1, 2017, and continued through December 31, 2019. We defined year one as November 1, 2017, to October 31, 2018, and year two as November 1, 2018, to December 31, 2019.

Intervention description

The evidence-based strategies highlighted in this QIP were based on previously published models [11,14] and focused on five key elements, namely, (1) accurate BP measurement, including repeating the BP assessment if the first BP was elevated; (2) timely follow-up (monthly follow-up in staff-led visits until the BP was controlled); (3) a treatment algorithm that prioritized once-daily, low-cost medications; (4) outreach to patients with elevated BP using an EHR-based registry; and (5) a communication curriculum highlighting building trusting relationships with patients. At the kick-off, practices were provided a hypertension change package (www.Cardi-OH.org/qip) developed by a team of experts (i.e., hypertension expert, primary care providers, QI expert, and health sociologist) based on prior successful hypertension regional efforts along with baseline EHR data process and outcome measures to guide them in developing their practice-specific SMART aims and in implementing these strategies.

The Medicaid MCPs also developed, tested, and implemented parallel QI interventions informed by discussions with the primary care practices and ODM for Medicaid enrollees seen at the practices. One of the key aspects of the project was having MCPs and primary care providers collaboratively conduct QI activities to benefit patients/enrollees. The iterative MCP Plan, Do, Study, Act (PDSA) differed by payer but focused on medication adherence including the provision of a 90-day supply of BP medications (versus the 30-day supply previously allowed) and outreach to Medicaid patients with elevated BP to re-engage them in care. MCPs also developed patient resource sheets and standardized order forms used by the practices to support at-home BP monitoring for Medicaid enrollees. While practice-level interventions included all patients, MCP interventions focused specifically on Medicaid enrollees.

Implementation description

We used the following implementation strategies: (1) QI coaching/practice facilitation; (2) audit and feedback; and (3) peer-to-peer learning. The Hypertension QIP initially had an in-person kick-off in October 2017 to review the hypertension change package (www.Cardi-OH.org/qip) comprising practice-level interventions and to begin PDSA planning with the practices. After the kick-off, practice facilitators were encouraged to meet monthly with practices to assist with their QI efforts. ODM also paired a Medicaid MCP with each practice and encouraged biweekly meetings to address non-clinical barriers such as transportation to improve BP control. The project team (CWRU, ODM, GRC, QI consultant) met weekly regarding the project challenges and to plan the monthly webinars or Action Period calls which reviewed EHR data on progress, ensured peer-to-peer learning among practices and MCPs, and provided QI and/or clinical didactics from experts. While data were collected at the individual level, they were rolled up to the practice level for audit and feedback purposes. De-identified, aggregated, practice-level EHR data for the overall patient population and stratified by race/ethnicity were shared every two weeks with primary care practices and payers to support continuous QI efforts.

Measures

We included practice-level process and outcome measures using EHR data. We used statistical process control charts to share measures with practices for QI. The primary outcome measure was the percentage of adult patients with hypertension under BP control (defined as <140/90 mmHg) overall and stratified by race/ethnicity [non-Hispanic Black (NHB) and non-Hispanic White (NHW)]. We defined BP control as <140/90 mmHg using the office BP readings reported in the EHR as the national quality metrics for value-based payment models were using this definition. Process measures included (1) repeat BP: percentage of hypertensive adults with a repeat BP at the same office visit if the first BP was elevated (>140/90 mmHg); (2) timely follow-up scheduled: percentage of hypertensive adults with elevated BP with a scheduled follow-up visit within 35 days; and (3) timely follow-up attended: percentage of adults with hypertension with an attended follow-up visit within 35 days.

Data collection and management

We extracted data from the EHR for patient demographics and selected clinical variables. For individuals enrolled in Ohio Medicaid, we also used Medicaid enrollment and claims data to extract data on BP medication adherence. Everyone was assigned a unique Patient ID, which allowed records for the same individual to be identified across time. For individuals enrolled in Medicaid, the Medicaid ID allowed the identification of matching records in the Medicaid claims files. If the Medicaid ID was missing in the EHR and the patient did not have another insurance type listed, we used patient name, date of birth, visit date, and street address and zip code to link the patient EHR and Medicaid claims data (total linkage rate of 83%). For data linkage, we used Link Plus, a probabilistic record linkage program developed by the Centers for Disease Control and Prevention [18].

For the modeling analysis, the primary outcome was BP control, defined, at the individual level, and within each of the periods defined above for baseline, year one, and year 2, as the proportion of primary care visits with BP control (<140/90 mmHg) exceeding 50% during that period. Independent variables from the EHR were based on the first non-missing value. Variables included age (18-24, 25-44, 45-64, or 65+ years), sex (male or female), race/ethnicity (NHW, NHB, Hispanic, Asian, and/or Other/Unknown), body mass index (BMI) [underweight (BMI <18.5 kg/m^2^), normal (BMI = 18.5-24.9 kg/m^2^), overweight (BMI = 25.0-29.9 kg/m^2^), or obese (BMI >30.0 kg/m^2^)], smoking status (current, former, or never), and insurance status (Medicare, Medicaid, Dually eligible Medicare/Medicaid, Private, or Uninsured). Hispanic ethnicity was coded independent of race. Three comorbid conditions were extracted from the EHR, namely, diabetes, depression, and congestive heart failure. The site of care was labeled as A through H. The Medicaid category was classified as “Medicaid Continuous” for those continuously enrolled during the project period and “Medicaid Non-continuous” for all other Medicaid enrollees. For medication adherence, we defined the medication possession ratio (MPR) as the total medication days supplied minus the medication days supplied from the last fill divided by the total number of days from the first fill date to the last fill date [19]. If an individual had more than one BP medication, the MPR was calculated separately for each BP medication and averaged across all BP medications. Consistent with previous studies, we defined “adherent” as an MPR >0.8 (80% adherent) [19-21].

Data analysis

We used two approaches to evaluate the impact of the QIP over time. First, we used statistical process control (SPC) charts to demonstrate changes in the following EHR-based process and outcome measures for all patients seen regardless of insurance status: (1) BP control overall and stratified by race/ethnicity; (2) repeat BP; and (3) timely follow-up scheduled and attended (described under measures). The three main components of an SPC chart are a central line (CL) for the average, a lower control line (LCL) for the lower control unit, and an upper control line (UCL) for the upper control unit.

Second, we modeled the data to evaluate BP changes over time adjusted for covariates. The relationship between the QIP intervention and the primary outcome, BP control, was modeled using weighted generalized estimating equations. The first set of models used data from all patients in our study population; the second group of models used only data from those continuously enrolled in the Ohio Medicaid program between January 1, 2017, and December 31, 2019, to include medication adherence.

The models estimate the log odds that BP would be controlled at an average visit during each cross-sectional intervention period (i.e., baseline, year one, and year two as defined earlier). We weighted the number of visits patients had during each study period and stratified all models by race/ethnicity as the reduction in racial inequity was one of our aims. Due to small sample sizes in racial and ethnic groups other than NHW and NHB, all models were restricted to individuals who self-identified as belonging to one of these two groups. All models were adjusted for the independent variables listed above except for medication adherence; this variable was only available for Medicaid patients. To assess the validity of the model results, a sensitivity analysis was performed using only the cohort of patients who had visited in all three intervention periods. We exponentiated the log odds to odds ratios (ORs) for ease of interpretation. Data management and modeling were performed using R version 3.6 [22] and SAS version 9.4 (SAS Institute Inc., Cary, NC, USA) [23].

## Results

Study population characteristics

Table 2 presents the characteristics of the study population. In total, there were 17,672 unique participants seen at 93,781 visits across all practices for the entire study period. Most patients (79%) were over 45 years of age, and about half were women (55%). Half of the study population identified as NHB, 45% as NHW, 2% as Hispanic, and 3% as other. Slightly over half (56%) were obese with a BMI of 30 or greater, and about one-third were current smokers. Participants were enrolled in Medicaid (28%), Medicare (26%), private insurance (33%), and self-pay or uninsured (13%). Demographic characteristics changed little from the baseline over the study period.

**Table 2:** Study population characteristics overall and by project period. Note: First non-missing value during each intervention period is used for all covariates. ^1^: Total number of unique patients who had ≥1 visit during the time period of interest. ^2^: Defined as a proportion of visits within the study phase with BP control (<140/90 mm Hg) exceeding 50%. ^3^: Hispanic race classified if the race or ethnicity was Hispanic. ^4^: Data missing for 223 individuals. ^5^: Data missing for 92 individuals. ^6^: Defined as a medication possession ratio ≥0.80; continuously enrolled Medicaid patients filling ≥1 medication twice. Data missing for 88 individuals.

	Total^1^			Pre-intervention				Year 1			Year 2	
	n	n %	Total^2^		Col %	% Control^2^	Total^1^	Col %	% Control^2^	Total^1^	Col %	% Control^2^
Total												
Participants	17,672		8,839			46.5%	11,436		49.3%	12,460		50.9%
Visits	93,781		21,845			53.3%	32,852		56.9%	39,084		57.6%
Site												
A	1,646	9.3	281		3.2	43.8%	1,159	10.1	34.8%	1,269	10.2	38.4%
B	559	3.2	203		2.3	33.0%	261	2.3	33.0%	362	2.9	41.2%
C	1,380	7.8	500		5.7	62.2%	1,081	9.5	67.9%	1,106	8.9	74.6%
D	3,808	21.5	2,067		23.4	40.8%	2,393	20.9	47.1%	2,554	20.5	49.9%
E	4,961	28.1	2,902		32.8	48.1%	3,149	27.5	51.2%	3,416	27.4	48.4%
F	705	4.0	409		4.6	38.9%	470	4.1	49.4%	542	4.3	55.5%
G	3,309	18.7	1,744		19.7	52.4%	2,075	18.1	54.7%	2,485	19.9	54.9%
H	1,304	7.4	733		8.3	40.4%	848	7.4	36.7%	726	5.8	39.8%
Age (years)												
18–24	191	1.1	57		0.6	38.6%	84	0.7	51.2%	105	0.8	59.0%
25–44	3,445	19.5	1,481		16.8	41.5%	1,873	16.4	42.6%	2,087	16.7	43.3%
45–64	9,388	53.1	4,902		55.5	47.3%	6,064	53.0	50.8%	6,462	51.9	52.5%
65+	4,648	26.3	2,399		27.1	48.1%	3,415	29.9	50.4%	3,806	30.5	52.2%
Sex												
Female	9,700	54.9	4,922		55.7	48.1%	6,406	56.0	51.2%	6,941	55.7	51.9%
Male	7,972	45.1	3,917		44.3	44.4%	5,030	44.0	46.9%	5,519	44.3	49.7%
Race^3^												
Hispanic	408	2.3	206		2.3	49.0%	253	2.2	48.6%	288	2.3	52.4%
Non-Hispanic Asian	140	0.8	67		0.8	46.3%	87	0.8	47.1%	96	0.8	53.1%
Non-Hispanic Black	8,845	50.1	4,429		50.1	43.4%	5,761	50.4	44.0%	6,285	50.4	45.5%
Non-Hispanic White	7,892	44.7	3,957		44.8	49.8%	5,108	44.7	55.3%	5,529	44.4	56.9%
Other/unknown	387	2.2	180		2.0	46.7%	227	2.0	52.0%	262	2.1	52.3%
Body mass index (BMI)^4^												
Underweight (BMI <18.5)	210	1.2	102		1.2	51.0%	121	1.1	47.1%	127	1.0	48.0%
Normal weight (18.5 ≤ BMI < 25)	2,848	16.1	1,357		15.4	45.4%	1,774	15.5	48.4%	1,916	15.4	49.3%
Overweight (25 ≤ BMI < 30)	4,425	25.0	2,180		24.7	48.1%	2,828	24.7	49.1%	3,102	24.9	50.8%
Obese (BMI ≥30)	9,966	56.4	5,020		56.8	46.0%	6,538	57.2	49.9%	7,146	57.4	51.6%
Smoking^5^												
Current	6,090	34.5	2,871		32.5	44.9%	3,797	33.2	45.8%	4,041	32.4	48.2%
Former	4,876	27.6	2,736		31.0	50.0%	3,257	28.5	54.7%	3,523	28.3	55.3%
Never	6,614	37.4	3,180		36.0	44.9%	4,287	37.5	48.4%	4,790	38.4	50.2%
Comorbidities												
Congestive heart failure	1,248	7.1	717		8.1	50.2%	807	7.1	50.1%	823	6.6	52.7%
Depression	2,855	16.2	1,500		17.0	48.2%	1,950	17.1	49.7%	2,066	16.6	52.8%
Diabetes	3,952	22.4	2,313		26.2	45.0%	2,738	23.9	49.9%	2,726	21.9	52.5%
Adherence^6^												
Yes	1,927	10.9	1,393		15.8	53.5%	1,587	13.9	58.0%	1,591	12.8	59.4%
Insurance category												
Medicaid - continuous	4,176	23.6	2,764		31.3	49.6%	3,163	27.7	53.5%	3,213	25.8	55.5%
Medicaid - non-continuous	827	4.7	345		3.9	43.5%	422	3.7	37.7%	411	3.3	39.9%
Medicare	4,527	25.6	2,138		24.2	47.8%	3,040	26.6	49.7%	3,382	27.1	52.0%
Private	5,872	33.2	2,104		23.8	44.3%	3,650	31.9	49.0%	4,528	36.3	50.4%
Self-pay	2,270	12.8	1,488		16.8	42.5%	1,161	10.2	42.5%	926	7.4	38.2%

Unadjusted primary outcome and process measures

**Figure 2:**
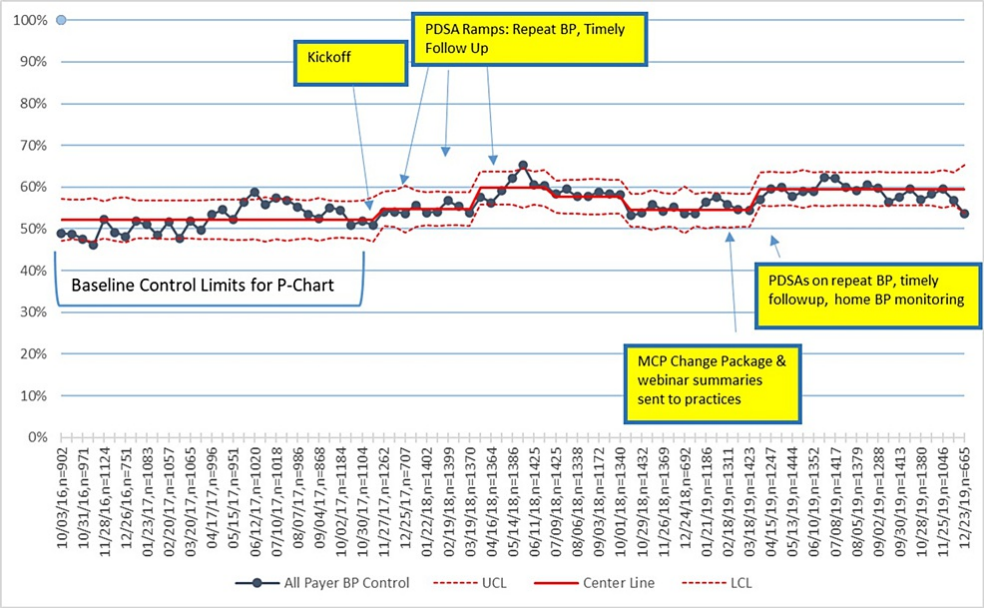
Control chart of BP control. PDSA = Plan, Do Study, Act; MCP = Managed Care Plans; BP = blood pressure; UCL = upper center line; LCL = lower center line

**Figure 3:**
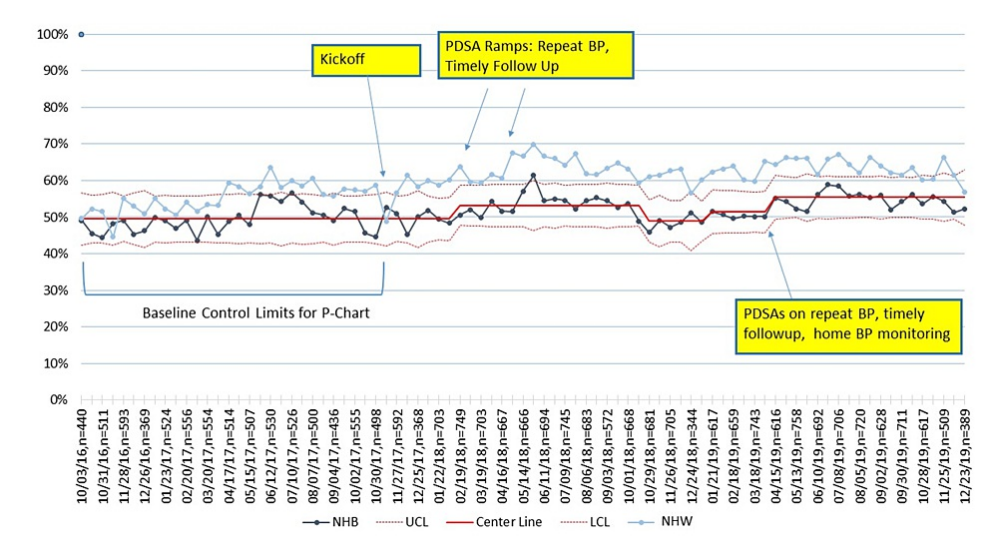
Control charts of BP control by race/ethnicity. PDSA = Plan, Do Study, Act; MCP = Managed Care Plans; BP = blood pressure; NHB = Non-Hispanic Black; NHW = Non-Hispanic White; UCL = upper center line; LCL = lower center line

**Figure 4:**
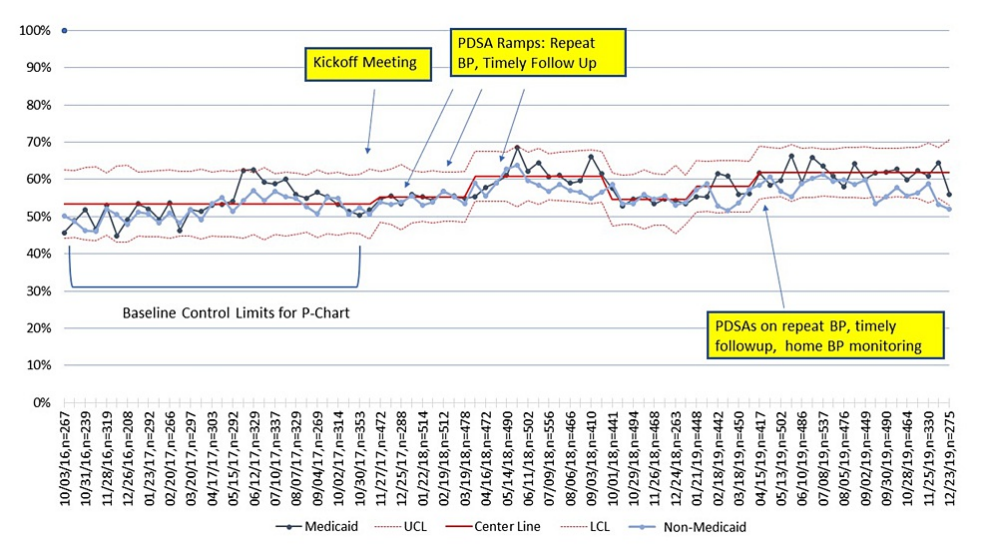
Control charts of BP control by Medicaid status. PDSA = Plan, Do Study, Act; MCP = Managed Care Plans; BP = blood pressure; UCL = upper center line; LCL = lower center line

**Figure 5:**
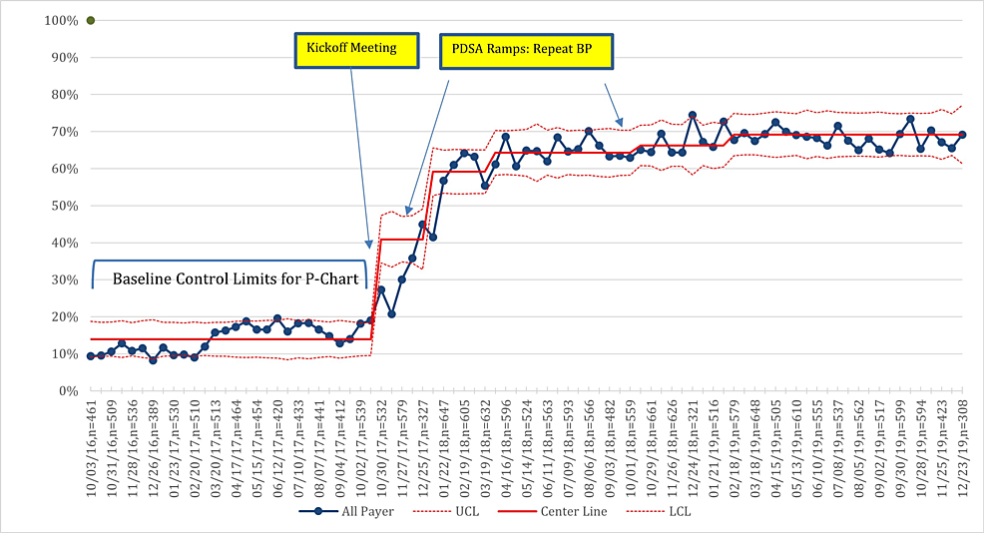
Percentage of hypertensive patients with a repeat BP if the first BP is elevated (>140/90 mmHg). PDSA = Plan, Do Study, Act; BP = blood pressure; UCL = upper center line; LCL = lower center line

**Figure 6:**
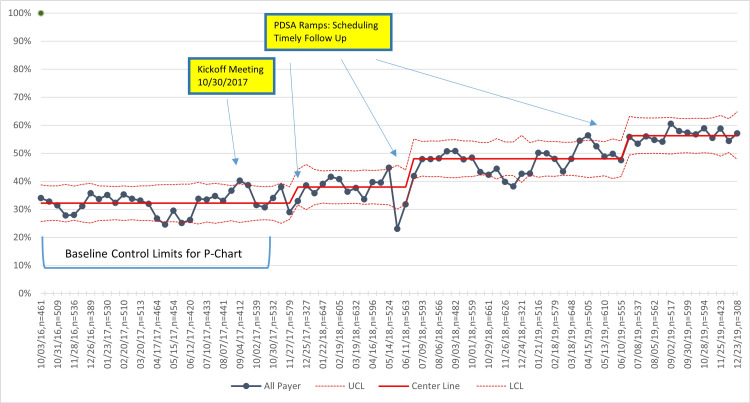
Percentage of hypertensive patients with elevated BP (>140/90 mmHg) with a scheduled follow-up visit within one month. PDSA = Plan, Do, Study, Act; BP = blood pressure; UCL = upper control limit; and LCL = lower control limit

**Figure 7:**
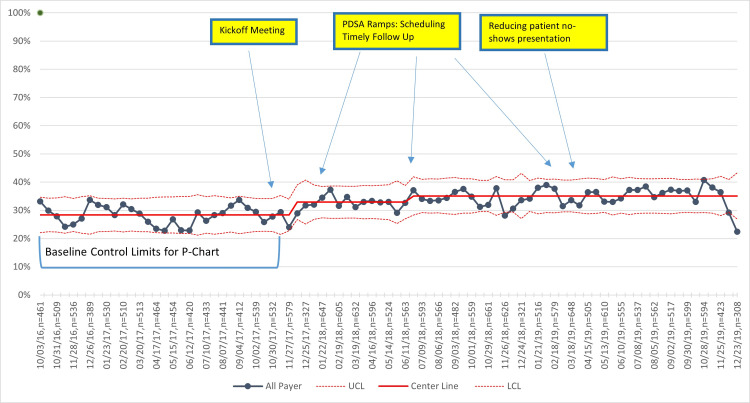
Percentage of hypertensive patients (whether BP elevated or not) who attended a follow-up visit within 30 days. PDSA = Plan, Do, Study, Act; BP = blood pressure; UCL = upper center line; LCL = lower center line

For all practices combined, the percentage of participants with controlled BP increased from 52% at baseline to 60% in the last quarter of 2019, with variation occurring during the study period (Figure 2). BP control improvements over the same period were 5% and 10%, respectively, for NHB and NHW patients (Figure 3), and 9% and 7%, respectively, for Medicaid versus non-Medicaid patients (Figure 4). BP control rates started lower for NHBs and remained lower than NHWs through the end of the project period (Figure 3). All three process measures (repeat BP, timely follow-up visit scheduled, and timely follow-up visit attended) improved over time (Figures 5-7).

Adjusted models for BP control improvement

**Figure 8:**
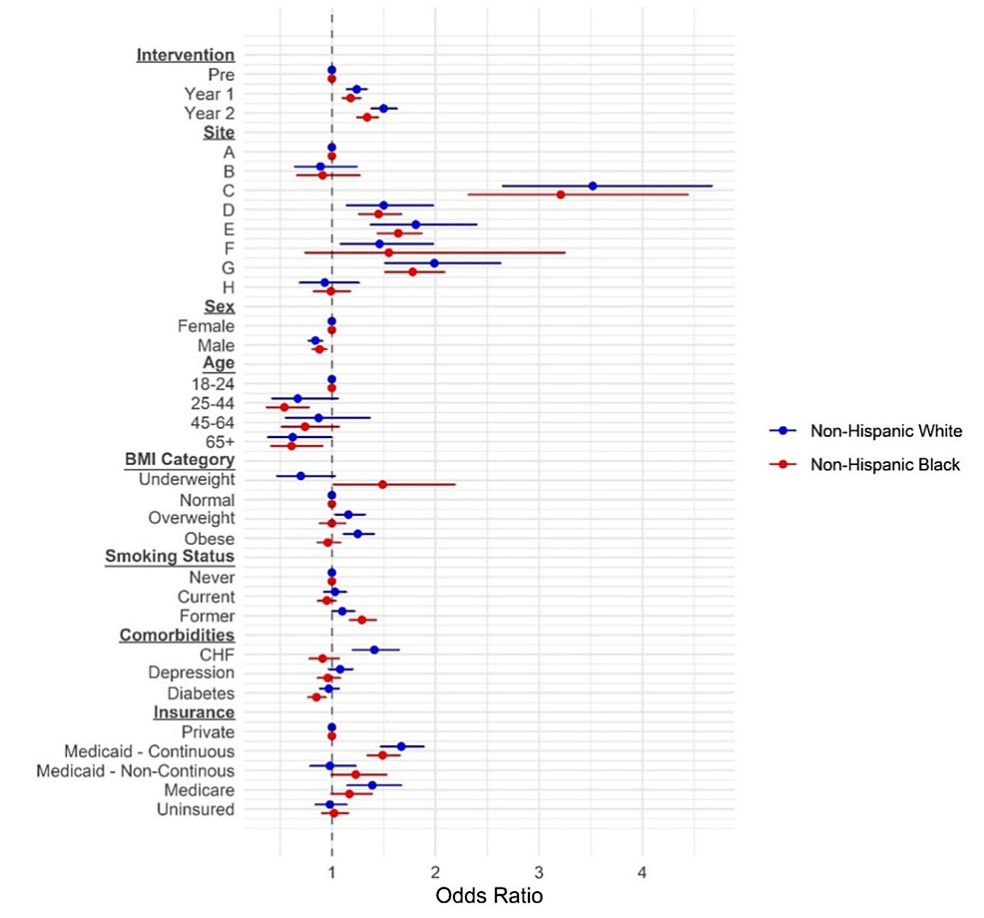
Odds of blood pressure control for various subgroup associations. BMI = body mass index; CHF = congestive heart failure

Figure 8 shows the results from the multivariable models. For NHWs and NHBs, the odds of achieving BP control were higher in year one and year two relative to baseline, adjusting for QIP site, age, sex, BMI, smoking status, comorbidities, and insurance status. In NHWs, the odds of achieving BP control in year one and year two were 1.24 times (95% confidence interval: 1.14, 1.34) and 1.5 times (1.38, 1.63) higher relative to baseline, respectively. In NHBs, the odds for years one and two were 1.18 times (1.10, 1.27) and 1.34 times (1.24, 1.45) higher relative to baseline, respectively. Most sites had significant improvements in overall BP control in year two compared to baseline with variation by the site (range in OR for sites which improved, 1.17 to 2.36) (Table 3). These improvements occurred in NHB and NHW populations, although significance decreased at sites with smaller patient populations. In a sensitivity analysis, adjusted models showed similar results for a fixed cohort of the same patients over time (Table 4).

**Table 3:** Percentage of visits with BP controlled for adults with hypertension by site and intervention period. *: Denotes observed values; % of visits where BP <140/90 mmHg. Generalized estimating equations model if the average visit was controlled. BP = blood pressure; OR = odds ratio; N = number; CI = confidence interval

Site	Pre-intervention			Year 1				Year 2			
	% <140/90 mm Hg*	OR	N	% <140/90 mm Hg*	OR	95% CI	N	% <140/90 mm Hg*	OR	95% CI	N
A	47.5	1	272	42.7	0.78	(0.58, 1.04)	1,112	46.3	0.99	(0.74, 1.33)	1207
B	36.6	1	187	40.2	1.22	(0.78, 1.91)	236	42.9	1.57	(1.04, 2.36)	341
C	66	1	483	72.5	1.42	(1.16, 1.73)	1,041	76.1	2.07	(1.66, 2.57)	1071
D	48.5	1	1964	55.4	1.32	(1.18, 1.48)	2,275	56.7	1.73	(1.53, 1.96)	2412
E	56.1	1	2821	58.7	1.21	(1.1, 1.33)	3,073	56.3	1.17	(1.06, 1.29)	3332
F	48.4	1	309	55.2	1.54	(1.16, 2.03)	349	59.9	2.36	(1.76, 3.16)	423
G	56.6	1	1581	56.7	1.1	(0.97, 1.24)	1,905	58	1.19	(1.05, 1.34)	2241
H	44.9	1	678	43.7	0.96	(0.78, 1.18)	788	46.2	1.22	(0.99, 1.51)	677

**Table 4:** Adjusted ORs of blood pressure control by race - cohort analysis. OR = odds ratio; CI = confidence interval; N = number, SE = standard error

	Non-Hispanic White					Non-Hispanic Black				
	OR	95% CI	SE	p	N	OR	95% CI	SE	p	N
Intercept	0.4	(0.13, 1.22)	0.57	0.1083	2,183	1.57	(0.18, 13.64)	1.1	0.6812	2,484
Intervention										
Pre	1	(1, 1)	0		2,183	1	(1, 1)	0		2,484
Year 1	1.32	(1.19, 1.46)	0.05	<0.0001	2,183	1.27	(1.16, 1.39)	0.05	<0.0001	2,484
Year 2	1.63	(1.46, 1.82)	0.06	<0.0001	2,183	1.36	(1.23, 1.5)	0.05	<0.0001	2,484
Site										
A	1	(1, 1)	0		16	1	(1, 1)	0		137
B	1.23	(0.48, 3.17)	0.48	0.6709	43	0.86	(0.43, 1.73)	0.36	0.6757	>20
C	5.02	(2.15, 11.74)	0.43	0.0002	300	2.35	(1.23, 4.5)	0.33	0.0096	30
D	1.89	(0.81, 4.42)	0.43	0.1427	504	1.46	(1.05, 2.03)	0.17	0.0229	596
E	2.59	(1.1, 6.08)	0.44	0.0291	372	1.67	(1.22, 2.3)	0.16	0.0016	1,199
F	1.97	(0.83, 4.66)	0.44	0.1245	200	2.67	(0.76, 9.34)	0.64	0.124	<11
G	2.48	(1.06, 5.81)	0.43	0.0363	636	1.65	(1.14, 2.39)	0.19	0.0084	307
H	1.28	(0.53, 3.08)	0.45	0.5815	112	0.95	(0.65, 1.38)	0.19	0.7799	182
Sex										
Female	1	(1, 1)	0		1,177	1	(1, 1)	0		1505
Male	0.82	(0.71, 0.94)	0.07	0.004	1,006	0.94	(0.81, 1.1)	0.08	0.4342	979
Age (years)										
18–24	1	(1, 1)	0		<11	1	(1, 1)	0		<11
25–44	0.87	(0.42, 1.82)	0.38	0.7168	>190	0.27	(0.03, 2.33)	1.09	0.2362	>390
45–64	1.07	(0.53, 2.18)	0.36	0.8423	1,183	0.34	(0.04, 2.91)	1.09	0.3269	1438
65+	0.71	(0.33, 1.5)	0.38	0.3689	796	0.37	(0.04, 3.19)	1.1	0.366	645
Body mass index category										
Underweight	0.81	(0.38, 1.7)	0.38	0.5712	20	1.09	(0.59, 2)	0.31	0.7816	21
Normal	1	(1, 1)	0		326	1	(1, 1)	0		351
Overweight	1.1	(0.88, 1.37)	0.11	0.4106	597	1.06	(0.83, 1.37)	0.13	0.6292	613
Obese	1.37	(1.11, 1.68)	0.11	0.0035	1,240	1.05	(0.82, 1.35)	0.13	0.6853	1499
Smoking status										
Never	1	(1, 1)	0	.	766	1	(1,1)	0		941
Current	1.09	(0.9, 1.31)	0.09	0.3751	657	1.13	(0.94,1.36)	0.09	0.1936	763
Former	1.05	(0.89, 1.23)	0.08	0.5805	760	1.23	(1.03,1.46)	0.09	0.0203	780
Comorbidities										
Congestive heart failure	1.32	(1.02, 1.72)	0.13	0.0355	168	0.85	(0.62,1.16)	0.16	0.3108	179
Depression	1.03	(0.86, 1.23)	0.09	0.7235	467	0.88	(0.7,1.11)	0.12	0.2784	332
Diabetes	0.88	(0.75, 1.03)	0.08	0.1105	640	0.73	(0.6,0.87)	0.09	0.0007	623
Insurance										
Private	1	(1, 1)	0		510	1	(1,1)	0		533
Medicaid - continuous	1.42	(1.16, 1.74)	0.1	0.0008	692	1.1	(0.89,1.36)	0.11	0.3618	1121
Medicaid - non-continuous	0.9	(0.59, 1.39)	0.22	0.6455	55	0.65	(0.38,1.1)	0.27	0.107	54
Medicare	1.17	(0.86, 1.6)	0.16	0.3105	719	0.74	(0.53,1.05)	0.18	0.0947	453
Uninsured	0.92	(0.69, 1.21)	0.14	0.5355	207	0.83	(0.64,1.07)	0.13	0.1488	323

Focusing on Medicaid patients only, we observed higher odds of achieving BP control in years one and two compared to baseline (Table 5). In NHWs, the odds of achieving BP control were 1.41 times (1.17, 1.69) and 1.55 times (1.28, 1.87) higher in years one and two, respectively, relative to baseline. In NHBs, the odds of achieving BP control were 1.24 times (1.08, 1.43) and 1.38 times (1.19, 1.59) higher in years one and two, respectively, relative to baseline. Among participants on Medicaid, being adherent to anti-hypertensive medications was associated with significantly higher odds of achieving BP control by 1.52 times (1.21, 1.91) in NHWs and by 1.85 times (1.57, 2.19) in NHBs.

**Table 5:** Adjusted ORs of blood pressure control by race for Medicaid enrollees. Note: Reference levels were not having comorbidity. Medicaid status was denoted as “yes” if the participant was continuously enrolled in Ohio Medicaid Program between January 1, 2017, and December 31, 2019. Medicaid adherence was measured between January 1, 2017, and December 31, 2019. OR = odds ratio; CI = confidence interval; SE = standard error; N = number

	Non-Hispanic White					Non-Hispanic Black				
	OR	95% CI	SE	p	N	OR	95% CI	SE	p	N
Intercept	0.36	(0.06, 2.07)	0.9	0.2505	1,140	0.28	(0.07, 1.2)	0.74	0.0875	1,958
Intervention										
Pre	1	(1, 1)	0		811	1	(1, 1)	0		1,283
Year 1	1.41	(1.17, 1.69)	0.09	0.0002	866	1.24	(1.08, 1.43)	0.07	0.0021	1,497
Year 2	1.55	(1.28, 1.87)	0.1	<0.0001	863	1.38	(1.19, 1.59)	0.07	<0.0001	1,535
Site										
A	1	(1, 1)	0		47	1	(1, 1)	0		348
B	0.51	(0.22, 1.17)	0.43	0.1098	36	0.83	(0.39, 1.74)	0.38	0.618	>20
C	3.28	(1.87, 5.74)	0.29	<0.0001	205	2.94	(1.54, 5.61)	0.33	0.0011	35
D	1.78	(1.04, 3.04)	0.27	0.0351	358	1.48	(1.14, 1.93)	0.14	0.0038	430
E	2.17	(1.25, 3.77)	0.28	0.0062	249	2.01	(1.59, 2.55)	0.12	<0.0001	815
F	1.54	(0.79, 3)	0.34	0.2031	56	2.88	(0.70, 11.83)	0.72	0.1431	<11
G	1.45	(0.76, 2.79)	0.33	0.2592	71	1.45	(0.94, 2.22)	0.22	0.0909	90
H	1.12	(0.62, 2.04)	0.31	0.71	118	1.04	(0.76, 1.43)	0.16	0.794	215
Sex										
Female	1	(1, 1)	0		637	1	(1, 1)	0		1,205
Male	0.78	(0.64, 0.97)	0.11	0.023	503	0.77	(0.65, 0.91)	0.09	0.002	753
Age (years)										
18–24	1	(1, 1)	0		<11	1	(1, 1)	0		<11
25–44	1.1	(0.21, 5.77)	0.85	0.9103	>160	0.95	(0.23, 3.85)	0.71	0.9421	>430
45–64	1.28	(0.25, 6.6)	0.84	0.7704	826	1.2	(0.30, 4.84)	0.71	0.7963	1,220
65+	0.83	(0.16, 4.4)	0.85	0.8259	141	0.98	(0.24, 4.01)	0.72	0.9805	290
Body mass index category										
Underweight	0.57	(0.24, 1.33)	0.43	0.1911	18	0.83	(0.39, 1.73)	0.38	0.6135	18
Normal	1	(1, 1)	0		172	1	(1, 1)	0		302
Overweight	0.87	(0.62, 1.21)	0.17	0.3982	258	1.17	(090, 1.53)	0.13	0.2277	431
Obese	1.27	(0.93, 1.72)	0.16	0.1285	692	1.06	(0.84, 1.34)	0.12	0.6281	1,207
Smoking status										
Never	1	(1, 1)	0		281	1	(1, 1)	0		592
Current	1.03	(0.79, 1.34)	0.14	0.8305	515	1.13	(0.93, 1.37)	0.1	0.2337	842
Former	1.33	(1.01, 1.76)	0.14	0.0432	344	1.62	(1.31, 2.01)	0.11	<0.0001	524
Comorbidities										
Congestive heart failure	1	(0.67, 1.5)	0.21	0.9851	82	0.84	(0.62, 1.15)	0.16	0.2872	136
Depression	0.99	(0.78, 1.27)	0.13	0.9604	290	0.91	(0.73, 1.13)	0.11	0.4016	369
Diabetes	0.94	(0.74, 1.2)	0.12	0.6422	330	0.9	(0.74, 1.10)	0.1	0.3017	450
Adherence										
Yes	1.52	(1.21, 1.91)	0.12	0.0003	799	1.85	(1.57, 2.19)	0.1	<0.0001	1,122

## Discussion

This paper describes the establishment of a unique statewide QI collaboration among ODM, Medicaid MCPs, QI consultants, academic schools of medicine, and primary care practices. In this statewide pilot hypertension QIP in high-volume Medicaid primary care practices, BP control improved overall and within specific populations over time. Process measures of repeat BP and timely follow-up visits scheduled and attended also improved over time. Although improvements in BP control were seen in both NHW and NHB populations, NHBs started with lower rates of BP control which persisted in comparison to NHWs during the project period. While BP control improved at most sites, there was variation in improvement by sites.

Since 1980, multiple studies have shown improvements in BP control within primary care practices across health systems or within regional collaboratives [11,12,14,24,25]. We identified only one other statewide initiative which specifically focused on improving hypertension control [26]. This latter study used two mechanisms for BP control improvement, namely, (1) a data and monitoring feedback program using EHR data or cards completed by providers to track cardiovascular risk factor control and medication treatment patterns; and (2) an Experts in Hypertension Seminar Series [26,27]. While successful, this model did not initially engage with payers, although it subsequently involved payers to provide additional payment to practices with hypertension specialists. Our statewide external QI infrastructure and hypertension QIP demonstrates another successful model which included an initial in-person kick-off, monthly QI practice facilitation, and monthly webinars (with practices and Medicaid MCPs to provide performance feedback and shared learning from QI efforts) to improve BP control. EHR data allowed for rapid access to clinical data every two weeks for continuous QI activities.

Our project has several limitations which are common with QI studies. First, we used a bundled intervention and did not collect data on all individual elements within the bundle. Therefore, we are unable to comment on the individual effect of each element. The process measures used were indirect measures; repeat BP likely represented a focus on accurate BP measurement more broadly whereas timely follow-up promoted medication intensification, medication adherence strategies, and dietary change. The standardized strategies and measures around medication adherence and home BP monitoring by the MCPs were developed near the end of the pilot hypertension QIP; therefore, we did not have these process measures during this initial pilot. Prior work in our region suggests that repeating the BP measurement can improve BP control by 36% [28], and medication intensification also plays an important role [29]. Second, while we advocated for more potent doses of thiazide-type diuretics, especially chlorthalidone, prescription patterns were based on pharmacy claims data making real-time prescription patterns less available for intervention. Third, we did not require patient engagement, although we did have practices and MCPs conduct Pareto charts where they involved patient voice in identifying barriers to care. Fourth, we lacked a comparison group of primary care practices that did not participate in the QIP to help determine whether changes in BP control would have occurred without the intervention. However, national Healthcare Effectiveness Data and Information Set BP control measures showed little improvement in BP control from 2017 to 2019 except in Medicare Health Maintenance Organizations, which increased slightly from 57% to 61%, indicating no major secular changes which would have led to BP control improvement without an active intervention [30].

Given the lack of inequity reduction in our hypertension QIP, our project team has discussed several future directions to test for reductions in BP control inequities. With a second set of primary care practices participating in the hypertension QIP, specific interventions are being tested, including (1) more aggressive advocacy for increased use of chlorthalidone and amlodipine for their greater BP-lowering potency in NHBs and longer half-life which are more forgiving of missed doses using claims data run charts. Inadequate dosing of diuretics is commonly seen, especially in NHB patients with apparent treatment-resistant hypertension [31,32]. In addition, our Medicaid claims data have demonstrated lower medication adherence in NHBs compared to NHWs (data not shown). Further exploration of this measure, enhanced availability of 90-day prescription refills, and addressing other barriers related to medication taking are essential [33,34]. (2) Continued emphasis on effective communication. (3) Use of tailored interventions such as community health workers to address health-related social needs at practices where inequities exist. (4) Continued emphasis on the bundled intervention at practices with high volumes of NHB patients to ensure access to high-quality care regardless of site. In addition, the literature suggests we need to think more broadly about rigorously evaluating the effectiveness of addressing community-level, place-based drivers of inequities, including structural racism, toxic stress, and social drivers of health to eliminate inequities in BP control [35].

The development of a new collaborative requires time for appropriate engagement and data collection across partners. Delays in obtaining legal agreements for EHR data made it challenging to obtain timely and accurate data for continuous QI. Furthermore, Medicaid MCPs and primary care practices required support and guidance to develop trusting relationships that would lead to useful partnered interventions with practices and patients. A 12-month planning period would allow more time for building trusting partnerships and clear roles between project partners, developing payer interventions, and ensuring data and feedback were ready at the start of the project.

## Conclusions

In summary, a statewide QI infrastructure and initial hypertension QIP partnering with the ODM, Medicaid MCPs, academic medical centers, QI consultants, and primary care practices is feasible and led to improved BP control. The use of EHR data, monthly QI practice facilitation, and monthly webinars are effective strategies to support statewide continuous QI efforts of practices and payers. Future research should (1) investigate a modified approach incorporating strategies for inequity reduction with more practices and payers while including a comparison group of non-intervention sites; (2) explore factors that make some sites improve BP control or reduce inequities more than others, including the impact of specific payer contributions; and (3) evaluate the sustainability of hypertension QI improvements over time.
